# *RRR*-α-Tocopherol Is the Predominant Stereoisomer of α-Tocopherol in Human Milk

**DOI:** 10.1093/cdn/nzy055

**Published:** 2018-06-15

**Authors:** Matthew J Kuchan, Christopher J Moulton, Roger A Dyer, Soren K Jensen, Karen J Schimpf, Sheila M Innis

**Affiliations:** 1 Discovery Research and Development, Abbott Nutrition, Columbus, OH; 2 Analytical Research and Development, Abbott Nutrition, Columbus, OH; 3 BC Children's Hospital Research Institute, Department of Pediatrics, University of British Columbia, Vancouver, Canada; 4 Department of Animal Science, Aarhus University, Tjele, Denmark

**Keywords:** human milk, lactation, vitamin E, α-tocopherol, stereoisomer, *RRR*-α-tocopherol, *all-rac*-α-tocopherol

## Abstract

**Background:**

The naturally occurring α-tocopherol (α-T) stereoisomer, *RRR*-α-tocopherol (*RRR*-α-T), is known to be more bioactive than *all-rac*-α-tocopherol (*all-rac*-α-T), a synthetic racemic mixture of 8 stereoisomers. There is widespread use of *all-rac*-α-T in maternal supplements.

**Objective:**

The aim of the study was to thoroughly describe the α-T stereoisomer profile of human milk.

**Methods:**

We measured the α-T stereoisomer profile in milk from 2 cohorts of women: a cohort of 121 women who provided milk on days 30 and 60 of lactation (study 1) and a separate cohort of 51 women who provided milk on days 10, 21, 71, and 120 of lactation (study 2).

**Results:**

*RRR*-α-T was the predominant stereoisomer (*P* < 0.0001) in all samples in both studies despite a large intrasubject range in total α-T (0.7–22 μg/mL). On average, *RRR*-α-T comprised 73–76% of total α-T, but average values for the synthetic stereoisomers were *RRS*, 8–14%; *RSR*, 6–8%; *RSS*, 5–6%; and the sum of *2S* stereoisomers (Σ*2S*), 3–5%. Despite the predominance of *RRR*-α-T, the sum of the synthetic stereoisomers comprised as much as 48% of total α-T. We calculated the ratio of *RRR* to the sum of the synthetic *2R* (*RRS* + *RSR* + *RSS*) stereoisomers (s*2R*) to assess the degree to which *RRR* is favored in milk*.* Consistent with discrimination among 2*R* stereoisomers in mammary tissue, *RRR*/s2*R* values ranged from 2.8 to 3.6, as opposed to the expected ratio of 0.33 if there was no discrimination. However, the *RRR* to s*2R* ratio did not correlate with milk α-T concentration, but both components of the ratio did.

**Conclusions:**

*RRR*-α-T is the predominant stereoisomer in human milk, concentrations of synthetic 2*R* stereoisomers were notable, and the relation between milk total α-T and stereoisomer profile is complex. Due to the wide range found in milk α-T stereoisomer profile, investigation into its impact on α-T status and functional outcomes in breastfed infants is warranted.

## Introduction

Vitamin E is an essential nutrient known to be particularly important in the nervous system, because deficiency in humans leads to ataxia and myopathy (1–3). After birth, infants acquire vitamin E from human milk derived via the maternal diet, or from infant formulas that are required to be supplemented with levels of vitamin E that meet infant requirements.

Vitamin E activity can be derived from 4 tocopherol structural isomers (α-, β-, γ-, and δ-) and the corresponding 4 tocotrienols (α-, β-, γ-, and δ-). However, of the structural isomers, only α-tocopherol (α-T) can fulfill human vitamin E requirements (4). Good food sources of vitamin E include vegetable oils, nuts, dark-green leafy vegetables, and egg yolk (4) in addition to fortified foods and dietary supplements. α-T is widely accepted to play an important role in protecting membrane-bound unsaturated FAs from oxidation through its potent lipid-soluble, chain-breaking antioxidant activity (5).

 α-T is well established as a component of human milk (6–16), which is known to contain markedly higher concentrations of α-T than its structural isomers, β-, γ-, and δ-tocopherol (6–8). The concentration of milk α-T is highest in colostrum and subsequently declines with stage of lactation (7–9, 11). This phenomenon has been reviewed elsewhere (15, 16). Notably, milk α-T concentrations are highly variable at each stage of lactation and are logically dependent on factors including maternal diet, metabolism, and supplement use.

Because there are 3 chiral carbons in the structure of α-T, synthetically manufactured α-T [*all-rac*-α-tocopherol (*all-rac*-α-T)] is a racemic mixture of all 8 possible stereoisomers equally divided among 4 *2R* (*RRR, RRS, RSR, RSS*) and 4 *2S* (*SSS, SSR, SRS, SRR*) stereoisomers (17). In contrast, naturally occurring α-T is synthesized by plants and exists as a single stereoisomer, *RRR*-α-tocopherol (*RRR*-α-T; 2*R*, 4′*R*, 8′*R*-α-T), commonly referred to as natural vitamin E. In humans, only the *2R* stereoisomers are considered to have biological activity; thus, *RRR*-α-T is believed to have 2 times more vitamin E activity than *all-rac*-α-T (17, 18). The hepatic α-tocopherol transfer protein (α-TTP) preferentially binds *2R* stereoisomers for incorporation into VLDL, resulting in degradation of the majority of *2S* stereoisomers from *all-rac*-α-T (18–20).

The α-T stereoisomer profile in human milk has not been thoroughly described despite the widespread use of *all-rac*-α-T in prenatal and maternal supplements. A recent 2-group proof-of-principle study in 89 women from our group showed that the α-T stereoisomer profile in human milk is differentially affected by *RRR*-α-T or *all-rac*-α-T supplementation (21). Milk stereoisomer profiles varied widely at study entry, with a higher average level of *RRR*-α-T compared with the synthetic stereoisomers. The well-accepted differences in vitamin E activity between *RRR*-α-T and *all-rac*-α-T might be particularly important because it is thought that mature human milk might not meet infant vitamin E requirements (16). Furthermore, recent research indicates that the infant brain preferentially contains *RRR*-α-T (22) and the human placenta discriminates in favor of *RRR*-α-T (23). Therefore, the purpose of the present study was to determine the α-T stereoisomer profile in human milk from a large cross-sectional study (24–27) and a longitudinal study involving milk samples collected up until day 120 of lactation (28).

## Methods

### Study design and subjects

Study 1 subjects (*n* = 271) were enrolled from the Vancouver, Canada, area and comprised a subset of a previously reported larger study (24–27). Subjects were breastfeeding mothers of healthy full-term infants extensively described elsewhere (24). Participants in the original study were asked to supply a breast-milk sample at both 30 d (±2 d) and 60 d (±2 d) of lactation. Of the 235 subjects who completed the original study (24), 192 supplied ≥1 milk sample and 121 subjects supplied both milk samples; the current report provides data on these 121 subjects. Subjects completed a food record at 16 and 36 wk of gestation for the previous 4 wk using an interview-administered FFQ, and average daily nutrient intake was averaged as previously described (24). In addition, subjects provided yes-or-no information on their use of prenatal or multivitamins during pregnancy (24). During the conduct of this study, all commercial maternal supplements in Canada contained *all-rac*-α-T. The University of British Columbia Children's and Women's Health Center Research Ethics Board approved all aspects of the study, and informed consent was obtained from all participants.

Study 2 subjects (*n* = 52) were enrolled in the Foods for Health Institute Lactation Study at the University of California, Davis (28). Subjects were breastfeeding mothers of healthy full-term infants and are extensively described elsewhere (28). Milk samples were collected at 10 (days 6–13), 26 (days 21–27), 71 (days 57–74), and 120 (days 106–130) d of lactation from 51 of the 52 participants. Participants received lactation support and training on proper sample collection from a lactation consultant. Half of the participants completed an online Muldoon Omega-3 FFQ containing 444 items (Modified Block 2006–Bodnar FFQ, 2006; NutritionQuest/Block Dietary Data Systems), including supplemental α-T intake, at study entry between weeks 34 and 38 of gestation (28). Intake of *all-rac*-α-T or *RRR*-α-T was not differentiated. In addition, the subjects provided yes-or-no information on vitamin supplement use during study intervals (in days; 0–35, 36–65, 66–106, and 107–120). The University of California, Davis, Institutional Review Board approved all aspects of the study, and informed consent was obtained from all participants.

### Milk sample collection

Study 1 subjects were asked to collect midmilk by feeding the infant for 3 min then interrupting the feeding to gently express milk into a bottle. Milk samples were stored in their home freezer at –20°C immediately after collection. Samples were transferred to the laboratory at the next study visit (∼4 wk later). Samples were stored in the laboratory at –80°C until analysis.

Study 2 subjects collected milk samples in the morning 2–4 h after feeding their infant according to a modified published method (29) involving milk collection from 1 breast using a Harmony Manual Breast pump (Medela, Inc.) by the participant. Participants fully pumped 1 breast into a storage bottle, inverted 6 times, separated it into 12-mL aliquots in 15-mL polypropylene tubes, and subsequently froze the sample in the kitchen freezer (–20°C). Samples were picked up, transported to the laboratory on dry ice, and stored at –80°C until analysis.

### Tocopherol analysis

α-T concentration was determined by HPLC after saponification and extraction into heptane as described previously (30). Briefly, 500 µL of milk was saponified in a mixture of ethanol, methanol, ascorbic acid (20% wt:vol), and potassium hydroxide-water (1:1 wt:vol) at 80°C for 30 min and subsequently cooled and extracted into 2 portions of 5 mL heptane. From the combined heptane extracts, 100 μL was injected into the HPLC. The flow rate was 3.0 mL/min, with mobile phase [heptane containing 2-propanol (3.0 mL/L) and degassed with helium]. The HPLC 100 × 4.6 mm Brownlee Spheri-5 Silica 5-µm column (Perkin-Elmer GmbH) was used with the Perkin Elmer, Series 200 system. Identification and quantification of the vitamins were obtained by comparison of retention times, as well as peak areas with external standards. The following extinction coefficient used for α-T was ${\rm{A}}_{1{\rm{cm}}}^{1\% } = 71.0$ at 294 nm in ethanol (96% vol:vol). Fluorescence detection was performed with an excitation wavelength of 290 nm and an emission wavelength of 327 nm. The HPLC system was a Perkin Elmer, Series 200.

The stereochemical composition of α-T was determined after methylation into the corresponding methyl ethers and subsequent separation by chiral HPLC [Chiralcel OD-H column; 250 × 4.6 mm, 5-µm particle size, cellulose tris (3,5-dimethylphenylcarbamate); Daicel Chemical Industries, Ltd.]. This method separates the 8 stereoisomers of α-T into 5 peaks (30). Results are reported as the ratio of the observed stereoisomer among 5 peaks, and concentration was determined by calculation of each stereoisomer concentration from total α-T. Data are expressed as micrograms per milliliter of sample.

### Sample size and statistical analysis

Because study 1 and study 2 were descriptive studies, power calculations were not used to determine sample size. Furthermore, to our knowledge, the α-T stereoisomer profile in human milk has not been thoroughly reported. All data were analyzed with GraphPad Prism version 5.04 for Windows (GraphPad Software; www.graphpad.com). To detect significant main effects and interactions between main factors, data were analyzed via 1-factor ANOVA, and when appropriate, differences between means were evaluated by Tukey's multiple-comparison test (GraphPad Prism). Means that differed significantly after a multiple-comparison test are denoted using unique letter subscripts (*P* < 0.05). To determine significant correlations between nutrient concentrations and age or other nutrients, data were analyzed via Pearson's correlation. Mean ratios [*RRR/RRS *+ *RSR *+ *RSS* (*RRR*/s2*R*)] were compared with the use of the Mann-Whitney test, and quartile contingencies were compared by using the chi-square test. Unless indicated otherwise, data are expressed as treatment means ± SEMs.

## Results

### Subjects

**Table 1** shows subject demographic characteristics for studies 1 and 2. In study 1, 121 subjects provided a milk sample at both day 30 and day 60, and 71 provided either the day 30 or the day 60 sample. In order to reduce the potential for sampling bias, the results presented are for the 121 women who provided both samples. In study 2, 51 women provided 4 samples each for a total of 204 samples, one each on days 10, 26, 71, and 120 of lactation. The populations were similar except for a directionally higher percentage of Chinese and “other” ethnicities and a lower level of education in study 1. Most of both study populations were multiparous; both study populations also had low rates of smoking and high rates of mother-reported supplemental vitamin use.

**TABLE 1:** Descriptive characteristics of study 1 and study 2 subjects

	Study design	
	Study 1 (cross-sectional)^1^	Study 2 (longitudinal)^2^
	(*n* = 192)	(*n* = 51)
Age,^3^ y	33 ± 3.7 (20–40)	32 ± 3.9 (25–42)
Ethnicity, *n* (%)		
White	143 (75)	46 (90)
Chinese	18 (9)	2 (4)
Hispanic	4 (2)	2 (4)
Other	27 (14)	1 (2)
Education, *n* (%)		
High school	7 (4)	NC^4^
Trade school or university	106 (55)	14 (27)
Post-graduate	79 (41)	37 (73)
Parity, *n* (%)		
1	77 (40)	12 (24)
>1	115 (60)	39 (76)
Smoking status (no), *n* (%)	185 (96)	51 (100)
Maternal weight,^3^ kg	63 ± 9 (47–86)	70 ± 12 (49–108)
Prenatal vitamin or multivitamin use, *n* (% yes)		
During pregnancy	174/187 (93)^5^	NC
Days 0–35	NC	46 (90)^6^
Day 36–65	NC	46 (90)
Day 66–106	NC	41 (80)
Day 107–120	NC	37 (73)

1 Subjects are a subset of a previously reported larger study enrolled from the Vancouver, Canada, area (24–27).

2 Subjects are from the Foods for Health Institute Lactation Study at the University of California, Davis (28).

3 Values are means ± SDs; range in parentheses.

4 NC, Not collected.

5 Five subjects did not respond. All prenatal supplements in Canada contained *all-rac*-α-tocopherol during the study.

6 US prenatal supplements contained either *all-rac*-α-tocopherol or *RRR*-α-tocopherol.

### Total α-T

**FIGURE 1:**
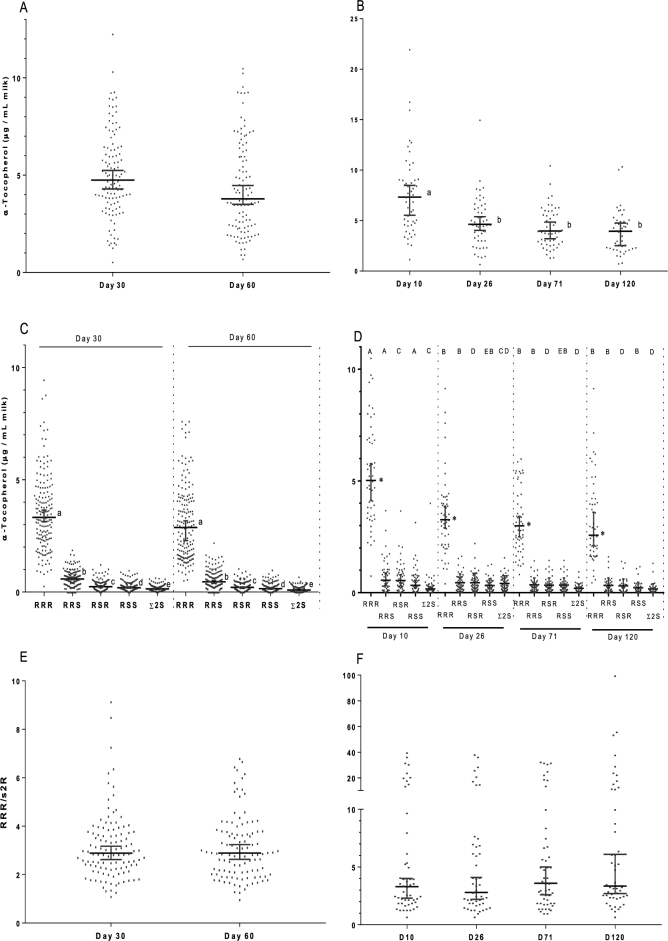
α-T profiles from study 1 and study 2 milk samples. (A) Study 1 milk total α-T concentrations; (B) study 2 milk total α-T concentrations; (C) Total α-T and α-T stereoisomer profiles in human milk; (D) α-T stereoisomer profile in study 2 milk samples; (E) *RRR*-to-s*2R* ratio (*RRR:RRS + RSR + RSS*) in study 1 milk samples; (F) *RRR*-to-s*2R* ratio in study 2 milk samples. Each data point is from a single milk sample collected from an individual subject. Horizontal bars show the median and 95% CI. Study 1 subjects were a subset of subjects from a previously reported larger study that enrolled participants from the Vancouver, Canada, area (24–27). Milk samples were collected at days 30 and 60 of lactation. Study 2 subjects were from the Foods for Health Institute Lactation Study at the University of California, Davis (28). Milk samples were collected at days 10, 26, 71, and 120 of lactation. ^a–e^Values differ, *P* < 0.0001; *values differ from all other stereoisomers at the same time point, *P* < 0.0001; ^A,B^values differ, *P* < 0.0001; ^C,D^values differ, *P* < 0.001; ^E^values differ, *P* < 0.01. D, day; s*2R*, sum of the synthetic *2R* (*RRS* + *RSR* + *RSS*) stereoisomers; α-T, α-tocopherol; *Σ2S*, *SSS* + *SSR + SRS + SRR*.

In study 1, median α-T concentration at day 30 of lactation was not different from that at 60 d of lactation (**Figure 1A**). Study 2 total α-T concentrations were higher at day 10 of lactation than at days 26, 71, and 120 of lactation (Figure 1B). Milk α-T concentrations were highly variable among subjects in both studies; values ranged from 0.6 to 12 μg/mL in study 1 and from 0.7 to 22 μg/mL in study 2. Values for Figure 1 are provided in **Supplemental Table 1**. Study 1 milk α-T concentration did not correlate with α-T intake collected at 32–36 wk (9.2 ± 3.80 mg/d; range: 1.5–25.4 mg/d) of gestation. Likewise, study 2 milk α-T concentration did not correlate with either dietary α-T intake or supplemental α-T intake collected between weeks 34 and 38 of gestation.

### α-T stereoisomers

In study 1, concentrations of *RRR*-α-T were significantly higher than those of each of the synthetic α-T stereoisomers at both days 30 and 60 of lactation (Figure 1C). The order of α-T stereoisomer concentrations at both time points was as follows: *RRR*-α-T >>* RRS*-α-T > *RSR*-α-T >* RSS*-α-T > sum of 2*S* stereoisomers (*Σ2S*) of α-T. At both days 30 and 60, the maximum value for each of the synthetic *2R* stereoisomers (*RRS*, 1.9; *RSR*, 1.2; *RRS*, 0.9 μg/dL) and *Σ2S* (0.7 μg/dL) was higher than the minimum value for *RRR*-α-T (0.3 μg/dL). Nonetheless, *RRR*-α-T was the most common stereoisomer in all study 1 milk samples; the mean percentage of total α-T measured as *RRR*-α-T was 73% for day 30 and 74% for day 60 (Supplemental Table 1).

Consistent with study 1 findings, *RRR*-α-T was the predominant α-T stereoisomer at each time point studied in study 2 (Figure 1D). The order of mean α-T stereoisomer concentration at each time point was as follows: *RRR*-α-T >>* RRS*-α-T = *RSR*-α-T =* RSS*-α-T = *Σ2S*-α-T. Consistent with a decrease in total α-T observed from day 10 to day 26 of lactation, each s*2R* stereoisomer, but not Σ2*S*, decreased from day 10 to day 26 of lactation. At each time point, the maximum value for each of the synthetic stereoisomers was higher than the minimum value for *RRR*-α-T. However, similar to study 1 findings, *RRR*-α-T was the most common stereoisomer in all study 2 milk samples; the mean percentage of total α-T measured as *RRR*-α-T ranged from 73% to 76% (Supplemental Table 1). Values for total α-T, the α-T stereoisomers, and the ratio of *RRR* to the s*2R* stereoisomers were consistent across studies. Milk *RRR*-α-T did not correlate with α-T intake in either study 1 or study 2; however, study 2 milk *RRR*-α-T at day 10 did positively correlate (*r*^2^ = 0.22, *P *< 0.0365) with supplemental intake (6.8 ± 5.4 mg/d; range: 0–20 mg/d) measured between weeks 34 and 38 of gestation.

### α-T stereoisomer ratios

In study 1, the median ratio of *RRR*-α-T to the sum of sythetic*2R* (s*2R*) stereoisomers (*RRR*/s2*R*) was ∼2.9 at both days 30 and 60 of lactation (Figure 1E). This ratio was highly variable, ranging from 1 to 9. A ratio could not be calculated for subjects who provided milk containing 100% of α-T as *RRR*-α-T or who had undetectable concentrations of ≥1 of the s*2R* stereoisomers (day 30, *n* = 6; day 60, *n* = 13). In study 2, the average *RRR*/s2*R* did not differ between the study days and was similar to those in study 1, ranging from 2.8 to 3.6 (Figure 1F). This ratio varied widely among study 2 individuals, ranging from 1 to 32.5 at each time point. Milk *RRR*-α-T positively correlated with milk total α-T in both studies (study 1: day 30, *r*^2^ = 0.911, *P *< 0.0001; day 60, *r*^2^ = 0.908, *P *< 0.0001; study 2: *r*^2^ values varied from 0.690 to 0.810, *P *< 0.0001 for each). As expected, s*2R* stereoisomers also positively correlated with milk α-T; however, the *RRR*/s2*R* did not in either of the studies (study 1: *r*^2^ = 0.002 for days 30 and 60; study 2: *r*^2^ ranged from 0.001 to 0.082).

### Quartile analysis of study 2 total α-T and *RRR*-α-T

To further understand the relation between total α-T and *RRR*-α-T over time, we grouped study 2 subjects into quartiles by their milk concentration of total α-T or *RRR*-α-T. We then determined if subjects differentially changed α-T and *RRR*-α-T quartile during the intervals from day 10 to day 26, from day 26 to day 71, and from day 71 to day 120. During each study interval, 29–35% of subjects remained in the same α-T quartile, but 76–80% remained in the same *RRR*-α-T quartile (**Table 2**; *P* < 0.0001).

**TABLE 2:** Quartile contingency analysis of total α-tocopherol and *RRR*-α-tocopherol in milk collected from days 10 to 120 of lactation^1^

	Quartile maintained		Quartile changed	
Comparison	α-Tocopherol	RRR-α-tocopherol	α-Tocopherol	RRR-α-tocopherol
Day 10 vs. Day 26	18 (35)	41 (80)^*^	33 (65)^*^	10 (20)
Day 26 vs. Day 71	15 (29)	39 (76)^*^	36 (71)^*^	12 (24)
Day 71 vs. Day 120	18 (35)	39 (76)^*^	33 (65)^*^	12 (24)

1 Each value is the number of subjects assigned to statistically determined quartiles; *n* (%). Each time frame was considered independently. Subjects (*n* = 52) were enrolled into the Foods for Health Institute Lactation Study and were from the University of California, Davis, area. Of the 52 subjects, 51 supplied milk samples (28). Chi-square test, *P* < 0.0001. ^*^RRR-α-tocopherol Quartile maintained > *RRR*-α-tocopherol Quartile Changed; α-Tocopherol Quartile changed > α-tocopherol Quartile maintained.

## Discussion

We report here that *RRR*-α-T is the predominant stereoisomer of α-T in human milk in 446 milk samples from 172 women from 2 North American locations. *RRR*-α-T was the most common single α-T stereoisomer in each of the milk samples studied, despite high intersubject variation in total α-T concentration and high self-reported use of dietary supplements containing *all-rac*-α-T. Our study describes inter- and intrasubject variation in the milk α-T stereoisomer profile in 2 cohorts, 1 cross-sectional and 1 longitudinal. We believe these data are novel because previous reports have thoroughly described the concentration of total α-T in human milk (6–16) but have not reported on α-T stereoisomer profile. The present data extend those from a recent report from our group that showed that average *RRR*-α-T was higher in milk than synthetic α-T stereoisomers in a smaller group of women after a 10-d supplement washout period. Understanding the α-T stereoisomer profile in human milk is particularly important considering that *RRR*-α-T has more vitamin E activity than *all-rac*-α-T (17–19).

A second key finding from the current research is that many milk samples contained appreciable concentrations of synthetic α-T stereoisomers despite the predominance of *RRR*-α-T. Interestingly, over time, fewer study 2 subjects changed quartile for *RRR*-α-T concentration than changed quartile for total α-T concentration. This suggests that the biological factors that determine milk *RRR*-α-T might be different than those that determine milk total α-T. Previous research has shown that milk *RRR*-α-T was decreased by supplementation of lactating women with *all-rac*-α-T (21). Because there are well-accepted differences in vitamin E activity between *RRR*-α-T and *all-rac*-α-T, the milk α-T stereoisomer profile might be particularly important considering that mature human milk may not meet infant vitamin E requirements (16). Consistent with the observation that *RRR*-α-T is favored in human milk, the human infant brain (22) and human placenta-fetal unit also appear to bio-discriminate in favor of *RRR*-α-T (23).

These observations are consistent with a previous report that showed a 2:1 *RRR*-α-T–to–*all-rac*-α-T ratio in milk from sows fed equal quantities of the 2 α-T sources (31). Consistent with that finding, we found milk samples that contained very high proportions of total α-T as *RRR*-α-T, including some samples that had 100% *RRR*-α-T. However, most milk samples contained an appreciable proportion of synthetic stereoisomers. Some samples from study 1 contained 50% of total α-T as synthetic stereoisomers, and in study 2 one individual produced milk that contained as much as 66% of total α-T as synthetic stereoisomers. It is not clear if this latter case reflects an unusual intake of supplemental *all-rac*-α-T or a polymorphism in α-TTP (32) or other factors.

Generally, our data indicate that *RRR*-α-T is favored among the 2R stereoisomers of α-T in human milk. We analyzed the *RRR* to s*2R* ratio as a means to assess this discrimination. This ratio was ∼3 in both studies, and the minimum ratio observed was ∼1. The same ratio in plasma has been reported to be 0.33 in women receiving oral *all-rac*-α-T (33). We suggest that the median ratio of ∼3 combined with minimum values >0.33 are consistent with breast tissue discrimination in favor of *RRR*-α-T. An alternate explanation is that the study populations consumed a high proportion of α-T from natural foods, as compared with that consumed from supplements. We cannot distinguish between these explanations; however, our qualitative dietary supplement intake data from study 2 indicate that median *RRR*/s2*R* values of 3 were unlikely to have been driven by diet alone.

Both milk *RRR*-α-T and s*2R* concentrations correlated positively with milk α-T concentration in both studies. In contrast, the milk ratio of *RRR* to s*2R* did not correlate with milk α-T concentration in either study. This suggests that milk total α-T concentrations are governed by factors different than those influencing the ratio of *RRR* to s2R. Milk α-T does not appear to be consistently influenced by habitual intake (16), a finding consistent with the current data. In contrast, the *RRR*/s2*R* might be influenced by the relative amount of α-T consumed from foods, which can naturally contain *RRR*-α-T or can be supplemented with *all-rac*-α-T, and by supplements that generally contain *all-rac*-α-T. In addition, the *RRR*/s2*R* might also be influenced by total α-T intake and the stereo-selectivity of α-TTP, for which polymorphisms have been reported (32). In addition, we found that the milk α-T composition from study 2 subjects was more likely to remain in the same *RRR*-α-T quartile than to remain in the same α-T quartile over the first 120 d of lactation. Taken together, our data suggest factors that determine the stereoisomer profile in human milk are complex.

A limitation of this report is that these studies were observational and therefore were not designed to identify the factors driving the variation observed in the milk α-T stereoisomer profile. In addition, detailed dietary and supplemental intakes of α-T were collected during gestation, but not lactation. The intake of foods supplemented with *all-rac*-α-T might have confounded milk *all-rac*-α-T because dietary intakes of *RRR*-α-T and *all-rac*-α-T were not distinguished. Without this information, it is not possible to deduce if the apparent bio-discrimination among α-T stereoisomers is influenced by the forms of dietary α-T. Interestingly, dietary intake of α-T during late pregnancy was not correlated with milk stereoisomer profile.

In conclusion, these data show a clear preferential accumulation of the naturally occurring α-T stereoisomer *RRR*-α-T in human milk. This was true of each of the 446 milk samples analyzed. Nonetheless, most samples contained detectable quantities of synthetic stereoisomers, and in some cases, their sum approached the concentration of *RRR*-α-T. Milk α-T stereoisomer profile may now be important to consider because mature human milk might not meet infant vitamin E requirements (16). Because *RRR*-α-T has more vitamin E activity than *all-rac*-α-T, variance in the human milk α-T stereoisomer profile could potentially accentuate shortfalls in infant vitamin E status. We believe these findings should stimulate further inquiry into the impact of human-milk α-T stereoisomer profile on infant vitamin E status.

## Supplementary Material

Supplement Table 1

## Notes

Supported by Abbott Nutrition.

Author disclosures: RAD, SKJ, and SMI, no conflicts of interest. MJK, CJM, and KJS are employed by Abbott Nutrition.

Supplemental Table 1 is available from the “Supplementary data” link in the online posting of the article and from the same link in the online table of contents at https://academic.oup.com/cdn/.

Abbreviations used:

*all-rac*-α-T: *all-rac*-α-tocopherol
*RRR*-α-T: *RRR*-α-tocopherol
s*2R*, sum of the synthetic: *2R* (*RRS* + *RSR* + *RSS*) stereoisomers, α-T, α-tocopherol; α-TTP, α-tocopherol transfer protein
Σ*2S*: sum of the 2S stereoisomers (*SSS* + *SSR* + *SRS* + *SRR*)
