# Supplementary material for: RRR-α-Tocopherol Is the Predominant Stereoisomer of α-Tocopherol in Human Milk
Source: Curr Dev Nutr. 2018 Jun 15;2(8):nzy055. doi: 10.1093/cdn/nzy055 (PMC6101621; doi:10.1093/cdn/nzy055)
Supplement: Supplement Table 1 [file nzy055_supplement_table_1.docx]

**Supplemental Table 1.** Total α-Tocopherol (α-T), α-Tocopherol Stereoisomer Profile, and *RRR*/s*2R* ratio in Milk Samples from Study 1 and Study 2^1,2^

|  |  | Study 1 | |  | Study 2 | | | |
| --- | --- | --- | --- | --- | --- | --- | --- | --- |
|  |  | Day 30^3^ | Day 60^3^ |  | Day 10 | Day 26 | Day 71 | Day 120 |
|  |  |  |  |  |  |  |  |  |
| *Total a-T* | μg/mL | 4.60 (4.56,5.36) | 3.98 (3.89,4.74) |  | 7.33 (6.51,8.73)^A^ | 4.63 (4.23,5.62)^B^ | 3.97 (3.81,4.90)^B^ | 3.95 (3.51,4.84)^B^ |
|  |  |  |  |  |  |  |  |  |
| *RRR-a-T* | μg/mL | 3.31 (3.29,3.87)^a^ | 2.89 (2.84,3.46)^a^ |  | 5.02 (4.70,5.96)^a,A^ | 3.26 (2.97,3.97)^a,B^ | 2.99 (2.81,3.61)^a,B^ | 2.57 (2.64,3.68)^a,B^ |
|  | % | 73, 50-100 | 74, 48-100 |  | 74, 34-97 | 73 (33-97) | 75, 46-97 | 76, 32-98 |
|  |  |  |  |  |  |  |  |  |
| *RRS-a-T* | μg/mL | 0.59 (0.59,0.72)^b^ | 0.47 (0.49,0.65)^b^ |  | 0.56 (0.54,0.96)^b,A^ | 0.33 (0.34,0.56)^b,B^ | 0.28 (0.27,0.44)^b,B^ | 0.26 (0.23,0.42)^b,B^ |
|  | % | 14, 6-25 | 14, 7-22 |  | 8, 0.7-23 | 8, 0-17 | 8, 0.4-19 | 8, 0-18 |
|  |  |  |  |  |  |  |  |  |
| *RSR-a-T* | μg/mL | 0.25 (0.25,0.33)^c^ | 0.21 (0.21,0.29)^c^ |  | 0.54 (0.51,0.89)^b,C^ | 0.34 (0.32,0.56)^b,D^ | 0.25 (0.25,0.43)^b,D^ | 0.25 (0.22,0.39)^b,D^ |
|  | % | 6, 1-12 | 6, 1-14 |  | 8, 0.7-17 | 8, 0-18 | 7, 0.6-17 | 7, 0-17 |
|  |  |  |  |  |  |  |  |  |
| *RSS-a-T* | μg/mL | 0.20 (0.22,0.29)^d^ | 0.15 (0.17,0.25)^d^ |  | 0.33 (0.37,0.68)^b,A^ | 0.24 (0.24,0.41)^b,EB^ | 0.25 (0.25,0.43)^b,EB^ | 0.15 (0.15,0.29)^b,B^ |
|  | % | 5, 0.5-11 | 5, 0.2-14 |  | 6, 0.5-14 | 6, 0-15 | 6, 0.3-12 | 5, 0-13 |
|  |  |  |  |  |  |  |  |  |
| Σ2*S-a-T* | μg/mL | 0.14 (0.15,0.21)^e^ | 0.10 + 0.14 (0,0.7)^e^ |  | 0.17 (0.15,0.47)^b,C^ | 0.34 (0.32,0.51)^b,CD^ | 0.12 (0.14,0.25)^b,D^ | 0.11 (0.10,0.23)^b,D^ |
|  | % | 4, 0.2-11 | 4, 0.1-10 |  | 3, 0.2-13 | 5, 0.2-20 | 4, 0.2-12 | 4, 0.2-20 |
|  |  |  |  |  |  |  |  |  |
| *RRR*/s*2R*^4^ | Ratio | 2.88 (2.93,3.44) | 2.90 (2.90,3.40) |  | 3.30 (4.62,10.11) | 2.80 (4.12,9.28) | 3.60 (4.76,9.82) | 3.35 (5.49,15.57) |
|  |  |  |  |  |  |  |  |  |

^1^ Values are μg α-tocopherol/mL of milk and are presented as median (95% Confidence interval). For α-T stereoisomers, % of total α-T is presented as mean %, range. *RRR/*s*2R* (*RRR/RRS+RSR+RSS*) values are median (95% Confidence interval). Σ2*S* is the sum of *SRR+SRS+SSR+SSS*.

^2^ Study 1 subjects were enrolled from the Vancouver, Canada area; Month 1, n=121, and Month 2, n=121 (refs 24-27). Study 2, 52 subjects were enrolled from the University of California Davis area; the Foods for Health Institute Lactation Study, and 51 supplied milk samples (ref 28).

^3^ Month 1 and Month 2 total α-T or α-T stereoisomer values were not different.

^4^ Study 1 values reflect n=115 at Month 1, and n=108 at Month 2. Study 2 values reflect n=51 for Day 10, n= 49 for Day 26, n=51 for Day 71, and n=50 for Day 120. Missing values occurred due to an undetectable value for at least one of the 2R stereoisomers.

^a-e^ Stereoisomer values within column with unlike superscripts differ, P<0.0001.
